# Functional status change in older adults undergoing coronary artery bypass surgery

**DOI:** 10.1590/S1516-31802011000200008

**Published:** 2011-03-03

**Authors:** Márcio Niemeyer Martins de Queiroz Guimarães, Clineu de Mello Almada Filho

**Affiliations:** I MD, MSc. Physician, Quinta D'Or Hospital, Rio de Janeiro, Brazil.; II MD, MSc, PhD. Physician, Universidade Federal de São Paulo (Unifesp), São Paulo, Brazil.

**Keywords:** Aged, Disabled persons, Coronary disease, Coronary artery bypass, Patient admission, Hospitalization, Idoso, Pessoas com deficiência, Doença das coronárias, Ponte de artéria coronária, Admissão do paciente, Hospitalização

## Abstract

**CONTEXT AND OBJECTIVE:**

Increased life expectancy has resulted in growing numbers of elderly patients undergoing heart surgery. This study aimed to identify changes in functional status among older adults undergoing coronary artery bypass grafting.

**DESIGN AND SETTING:**

Prospective observational cohort study conducted at a level IV private hospital in Brazil.

**METHODS:**

Patients were assessed using the Katz and Lawton scales and the Functional Independence Measure before admission, at hospital discharge and one month after discharge. Repeated-measurement analysis of variance was used.

**RESULTS:**

Two patients died during hospitalization. Among the 31 patients included, the Functional Independence Measure ranged from 121.7 ± 7.4 (pre-admission) to 91.1 ± 20.5 (discharge) and 109.0 ± 21.7 (one month after discharge); the Katz scale from 5.92 ± 0.32 to 4.18 ± 1.04 and 5.13 ± 1.30; and the Lawton scale from 24.3 ± 4.6 to 12.8 ± 2.0 and 16.5 ± 4.6 (P = 0.0001). When subgroups with (18) and without (13) complications were compared, the Functional Independence Measure (P = 0.085) showed a trend, although not significantly, toward recovery one month after discharge. Delirium and blood transfusion were the intercurrent events found. There was a correlation between the scales and age (P = 0.008), APACHE II (P = 0.051), EuroSCORE (P = 0.064), intensive care unit stay (P = 0.024) and overall hospital length of stay (P = 0.040).

**CONCLUSION:**

The Functional Independence Measure proved to be a promising tool for monitoring the functional status of elderly patients undergoing coronary artery bypass grafting, especially in the subgroup with complications.

## Abstract (pt)

**CONTEXTO E OBJETIVO:**

Com o aumento da expectativa de vida tem-se visto incremento nas cirurgias cardíacas em idosos. Este estudo teve como objetivo identificar variações da capacidade funcional em idosos submetidos a cirurgia de revascularização miocárdica.

**TIPO DE ESTUDO E LOCAL:**

Estudo de coorte, prospectivo e observacional realizado em um hospital privado nível IV, Brasil.

**MÉTODOS:**

Trinta e três pacientes foram avaliados pelas escalas de Katz e Lawton pela Medida de Independência Funcional na pré-internação, alta hospitalar e um mês pós-alta. Análise de variância para medidas repetidas foi utilizada.

**RESULTADOS:**

Dois óbitos ocorreram durante a hospitalização. Entre os 31 pacientes incluídos, a Medida de Independência Funcional variou de 121,7 ± 7,4 (pré-internação) a 91,1 ± 20,5 (alta) e 109,0 ± 21,7 (um mês pós-alta); Katz de 5,92 ± 0,32 a 4,18 ± 1,04 e 5,13 ± 1,30; e Lawton de 24,3 ± 4,6 a 12,8 ± 2,0 e 16,5 ± 4,6 (P = 0,0001). Quando comparados subgrupos com (18) e sem complicações (13), a Medida de Independência Funcional (P = 0,085) apresentou tendência, embora não significativa, de recuperação um mês pós-alta. Delirium e transfusão sanguínea foram intercorrências encontradas. Houve correlação entre as escalas com idade (P = 0,008), APACHE II (P = 0,051), EuroSCORE (P = 0,064), e tempos de permanência na unidade de terapia intensiva (P = 0,024) e no hospital (P = 0,040).

**CONCLUSÃO:**

A medida de independência funcional mostrou-se promissora para o acompanhamento da funcionalidade de idosos após a cirurgia de revascularização miocárdica, destacando-se subgrupo com complicações.

## INTRODUCTION

Cardiovascular events are among the most common diseases leading to death worldwide, and they stand out as a major cause of mortality. Comparison between the mortality rates in the main Brazilian centers and those from other countries shows high overall and cardiovascular disease mortality rates.^1^

Considering the changes in life expectancy in association with demographic and epidemiological morbidity and mortality data,^2^ and taking into account coronary heart disease, coronary artery bypass grafting is often suggested for elderly coronary patients. Despite the complexity of patient care resulting from a higher incidence of chronic diseases and involvement of other organs, advances in cardiac surgery and intensive care have translated into lower morbidity and mortality among these patients.^3^

Evaluation of patients regarding their ability to perform activities of daily living before hospital admission has important prognostic value. An assessment of daily activities is a strong predictor of health outcomes for functional status and survival. This is especially true for patients who are incapable of performing activities of daily living on admission, thus underscoring the prognostic importance of investigating the patient's functional status before the onset of an acute event.^4^

Many instruments have been proposed for reporting functional status in a manner that is understandable and reproducible, with systematic data collection, in particular the Katz Basic Activities of Daily Living scale^5^ and the Lawton Instrumental Activities of Daily Living scale.^6,7^ Due to the complexity of everyday actions and the heterogeneity of the elderly population, the literature describes several approaches concerning functional tests, which are not always applicable to all situations.^8-14^ A common characteristic of these scales is that they evaluate individuals only with regard to their performance in motor activities, without assessing, for example, communication skills or cognitive and social factors.

The Functional Independence Measure is an instrument developed to assess disability in patients with functional impairments of various origins. It aims to assess the quantity of care that an individual requires in order to perform a series of motor tasks, as well as assessing cognitive activities of daily living.^15–19^

## OBJECTIVES

To assess functional status among patients aged 60 years or older who were admitted to the intensive care unit (ICU) following coronary artery bypass grafting, investigating:

1. The existence of change in the Functional Independence Measure, and in the Katz and Lawton scales, analyzed separately for subgroups with and without complications during hospitalization, and measured on three occasions: pre-admission, hospital discharge and one month after discharge.
2. The existence of correlations between changes (delta) in the three functional status rating scales over two observation periods: pre-admission to hospital discharge and pre-admission to one month after discharge, with regard to clinical variables and the ICU and overall hospital lengths of stay.

## METHODS

This study was conducted at a level IV private hospital, according to the classification criteria for hospitals within the Brazilian National Health System,^20^ with 220 beds, including ICUs and medical-surgical inpatient units. Between 5,000 and 8,500 patients seek the emergency department per month in this service. Admissions range from 650 to 900 per month.

The study protocol was approved by the Research Ethics Committee of Universidade Federal de São Paulo (Unifesp), Brazil (Report No. 0134/09), and by the Research Ethics Committee of Rede D'Or, Brazil (Protocol No. 199/09).

Patients meeting the inclusion criteria provided written informed consent and were assessed using the Functional Independence Measure, Katz scale and Lawton scale before admission, at hospital discharge and one month after discharge.

The following data were collected: age; sex; history of diseases/conditions; need for blood transfusion during surgery; length of extracorporeal circulation during surgery (duration of perfusion and anoxia) in minutes; APACHE II^21^ and EuroSCORE^22^ scores on admission to the ICU; length of postoperative mechanical ventilation in minutes; need for vasoactive amines (norepinephrine) within the first 24 hours after admission to the ICU; complications or events during hospitalization, such as hemorrhage, infections, arrhythmias, need for pacemakers, acute myocardial infarction, acute stroke and need for further surgery; ICU and overall hospital lengths of stay.

### Study design and conditions

Data were collected prospectively between January and December 2009. This longitudinal, observational cohort study included elderly coronary inpatients undergoing coronary artery bypass grafting. Patients were selected according to known risk factors and the risks of elective surgery, when meeting the criteria for inclusion in the study. The objective was to use the functional status rating scales chosen to classify elderly individuals (older than 60 years of age, as defined by the World Health Organization^23,24^) who had been hospitalized to undergo elective cardiac surgery.

### Inclusion criteria

The patients included were all candidates for elective coronary artery bypass grafting and were aged ≥ 60 years.

### Exclusion criteria

Patients were excluded if they were ≥ 60 years of age and candidates for cardiac surgery other than coronary artery bypass grafting, such as aortic valve replacement or vascular surgery; and if they were younger than 60 years and candidates for coronary artery bypass grafting.

### Statistical analysis

The sample size was calculated by assuming a proportional decline (allowing for the expectation of recovery) in functional status, as measured by the Functional Independence Measure^19^ at the times of pre-admission, hospital discharge and one month after discharge (deltas), and also including complications.

By assuming an estimated proportional change in the Functional Independence Measure of around 0.25 (25% proportional decline), with a confidence level of 95%, an acceptable difference of 0.15 and an expected loss of individuals of 5%, it was determined that an adequate sample should include 34 patients.^25^

Repeated-measurement analysis of variance was used to evaluate the behavior of the Functional Independence Measure and the Lawton scale over the three observation time points (pre-admission, hospital discharge and one month after discharge), and Bonferroni's correction for multiple comparisons (adjusted for three stages) was used to identify which time points differed from each other.

Single-factor (effect of the interaction "group versus time") repeated-measurement analysis of variance was used to investigate whether the progression over time differed between the subgroups (with and without complications).

Changes in the Katz scale over the three observation time points were analyzed using Friedman's analysis of variance and the respective multiple comparison test.^26^

Spearman's correlation coefficient was used to measure the degree of association of deltas from the three functional status rating scales with clinical variables and length of stay.

Data from repeated-measurement analysis of variance were log-transformed, due to the ordinal nature and lack of symmetry in the distribution of these measurements. The significance level was set at 5%. Statistical analyses were performed using the SAS 6.04 software (SAS Institute, Inc., Cary, United States).

## RESULTS

The participants were inpatients who met the inclusion criteria between January and December 2009. Seventy-nine patients underwent cardiac surgery; of these, 26 underwent surgery other than coronary artery bypass grafting and were excluded. Among the 53 coronary artery bypass patients, 20 were excluded from the study because they did not meet criteria such as age (< 60 years) and/or refused consent. The 33 remaining patients were evaluated using the three functional status rating scales. Two patients died during hospitalization and were excluded from the sample. Thus, the final study sample comprised 31 patients.

### Cohort characteristics

Among the final sample, 22 patients were male (71%). The mean age was 69.32 years (minimum, 60 years; maximum, 86 years; median age, 69 years). Only eight patients were younger than 65 years. The most prevalent chronic diseases and/or conditions were hypertension (93.5%), dyslipidemia and sedentary lifestyle (both 54.8%), and diabetes mellitus and angina (both 41.9%).

Table 1 presents the mean ± standard deviation and median (minimum-maximum) of clinical variables and length of stay (numerical variables). Table 2 describes events during hospitalization (complications or intercurrent events).

**Table 1.** Clinical variables and length of stay

Variable	Mean	SD	Median	Minimum	Maximum
Extracorporeal circulation (perfusion)	92.7	39.7	90	32	233
Extracorporeal circulation (anoxia)	68.5	25.4	60	24	141
EuroSCORE	5.1	2.7	6	1	11
Risk of death (%) - EuroSCORE	5.8	5.0	5.01	1.21	21.34
APACHE II	14.3	4.1	13	7	22
Risk of death (%) - APACHE II	10.9	8.7	8.3	2.2	34.7
Mechanical ventilation (min)	701.8	471.0	510	165	1600
Length of ICU stay (days)	8.6	10.9	6	2	50
Length of hospital stay (days)	21.2	29.9	10	4	124

SD = standard deviation; APACHE = Acute Physiology and Chronic Health Evaluation; ICU = intensive care unit.

**Table 2.** Description of events during hospitalization (n = 31)

Variable	n	%
Postoperative mediastinitis	0	0
Perioperative blood transfusion	12	38.7
Postoperative hemorrhage	1	3.2
Pacemaker	1	3.2
Postoperative atrial fibrillation/flutter	3	9.7
Surgery on admission	3	9.7
Stroke on admission	1	3.2
Urinary tract infection on admission	3	9.7
Pneumonia on admission	1	3.2
Tracheobronchitis on admission	1	3.2
Atelectasis on admission	1	3.2
Norepinephrine > 24 h	6	31.6
Catheter-related infection	1	3.2
Surgical wound infection after saphenous vein stripping	0	0
Osteomyelitis	0	0
Delirium	4	12.9

Most of the sample (87%) underwent at least three bypass operations (three vessels revascularized); one patient aged 77 years underwent revascularization with a saphenous vein graft and two patients aged 64 and 68 years underwent revascularization with saphenous vein and mammary artery grafts. None of these patients had surgical complications. The mean duration of mechanical ventilation did not exceed 12 hours, and the maximum duration was approximately 27 hours. The length of extracorporeal circulation is described in Table 1.

The most prevalent events included perioperative blood transfusion (38.7%), use of norepinephrine for more than 24 hours (31.6%) and delirium (12.9%). Urinary tract infection was the most common infection (9.7%), and atrial fibrillation and atrial flutter were the most prevalent arrhythmias (9.7%). One patient had a stroke (3.2%), and one patient required temporary transvenous pacemaker insertion (3.2%). A total of 18 patients had complications or intercurrent events (58%) (Table 2).

### Evolution of functional status

Tables 3, 4 and 5 present the mean ± standard deviation and median (minimum-maximum) of the three rating scales, at the three observation time points (pre-admission, hospital discharge and one month after discharge), and the corresponding descriptive level (P) of the statistical analysis.

**Table 3.** Longitudinal analysis on the Functional Independence Measure (FIM)

FIM	Mean	SD	Median	Minimum	Maximum	P value*	Significant differences†
Pre-admission	121.7	7.4	124	86	126	0.0001	Pre ≠ discharge
Hospital discharge	91.1	20.5	96	37	123		Pre ≠ one month
After one month	109.0	21.7	117	31	125		Discharge ≠ one month

SD = standard deviation

* repeated-measurement ANOVA (effect of time)

† Bonferroni's multiple comparisons, 5% level.

**Table 4.** Longitudinal analysis on the Lawton scale

Lawton scale	Mean	SD	Median	Minimum	Maximum	P value*	Significant differences†
Pre-admission	24.3	4.6	27	12	27	0.0001	Pre ≠ discharge
Hospital discharge	12.8	2.0	13	9	21		Pre ≠ one month
After one month	16.5	4.6	16	9	27		Discharge ≠ one month

SD = standard deviation

* repeated-measurement ANOVA (effect of time)

† Bonferroni's multiple comparisons, 5% level.

**Table 5.** Longitudinal analysis on the Katz scale

Katz scale	Mean	SD	Median	Minimum	Maximum	P value*	Significant differences†
Pre-admission	5.92	0.32	6	4.5	6	0.0001	Pre ≠ discharge
Hospital discharge	4.18	1.04	4.5	1.5	6		Pre ≠ one month
After one month	5.13	1.30	5.5	0	6		Discharge ≠ one month

SD = standard deviation

* Friedman's ANOVA

† Multiple comparisons based on Friedman's test, 5% level.

**Figure 1:**
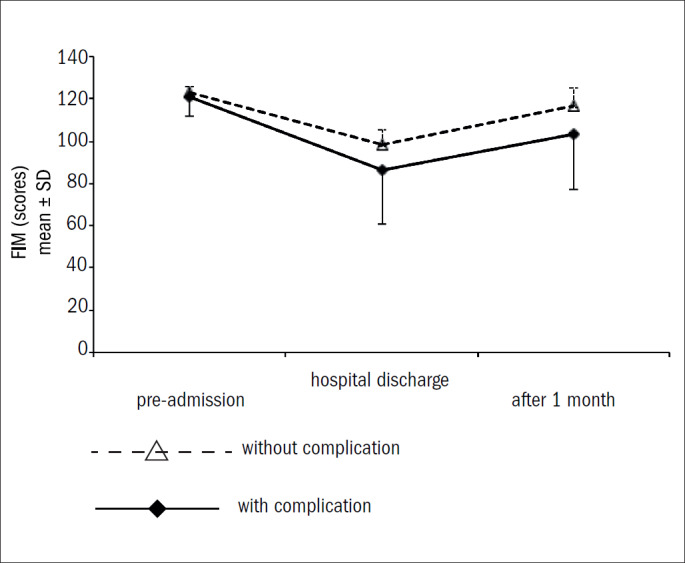
Functional Independence Measure (FIM) over time per complication subgroup.

Tables 6, 7 and 8 show the mean ± standard deviation and median (minimum-maximum) of the three rating scales, at the three observation time points (pre-admission, hospital discharge and one month after discharge) per subgroup (with and without complications), and the corresponding descriptive level (P) of the statistical analysis. Figure 1 shows the evolution of the Functional Independence Measure over time, per complication subgroup.

**Table 6.** Longitudinal analysis on the Functional Independence Measure per complication subgroup

Complications	Time point	Mean	SD	Median	Minimum	Maximum	P value*	Significant differences†	P value‡
Without (n = 13)	Pre-admission	122.8	3.3	124.0	116.0	126.0	0.0001	Pre ≠ discharge	0.085
	Hospital discharge	98.2	7.4	100.0	86.0	108.0		Discharge ≠ one month	
	After one month	116.8	8.3	120.0	97.0	125.0			
With (n = 18)	Pre-admission	120.9	9.3	123.5	86.0	126.0	0.0001	Pre ≠ discharge	
	Hospital discharge	85.9	25.2	95.5	37.0	123.0		Pre ≠ one month	
	After one month	103.3	26.5	113.0	31.0	124.0		Discharge ≠ one month	

SD = standard deviation

* Repeated-measurement ANOVA within each subgroup (effect of time)

† Bonferroni's multiple comparisons, 5% level

‡ Repeated-measurement ANOVA between two subgroups (effect of interaction: subgroup*time).

**Table 7.** Longitudinal analysis on the Katz scale per complication subgroup

Complications	Time point	Mean	SD	Median	Minimum	Maximum	P value*	Significant differences†	P value‡
Without (n = 13)	Pre-admission	6.00	0.00	6.00	6.00	6.00	0.0001	Pre ≠ discharge	0.12
	Hospital discharge	4.58	0.57	4.50	3.50	5.50		Discharge ≠ one month	
	After one month	5.65	0.47	6.00	4.50	6.00			
With (n = 17)	Pre-admission	5.91	0.36	6.00	4.50	6.00	0.0001	Pre ≠ discharge	
	Hospital discharge	4.03	1.08	4.00	1.50	6.00		Pre ≠ one month	
	After one month	5.03	1.07	5.50	2.50	6.00		Discharge ≠ one month	

SD = standard deviation

* Repeated-measurement ANOVA within each subgroup (effect of time)

† Bonferroni's multiple comparisons, 5% level

‡ Repeated-measurement ANOVA between two subgroups (effect of interaction: subgroup*time).

**Table 8.** Longitudinal analysis on the Lawton scale per complication subgroup

Complications	Time point	Mean	SD	Median	Minimum	Maximum	P value*	Significant differences†	P value‡
Without (n = 13)	Pre-admission	25.23	3.14	27.00	16.00	27.00	0.0001	Pre ≠ discharge	0.74
	Hospital discharge	13.08	0.95	13.00	12.00	15.00		Pre ≠ one month	
	After one month	17.23	3.03	16.00	13.00	23.00		Discharge ≠ one month	
With (n = 18)	Pre-admission	23.67	5.42	26.50	12.00	27.00	0.0001	Pre ≠ discharge	
	Hospital discharge	12.67	2.52	13.00	9.00	21.00	Pre ≠ one month		
	After one month	16.00	5.52	14.50	9.00	27.00	Discharge ≠ one month		

SD = standard deviation

* Repeated-measurement ANOVA within each subgroup (effect of time)

† Bonferroni's multiple comparisons, 5% level

‡ Repeated-measurement ANOVA between two subgroups (effect of interaction: subgroup*time).

Log transformation resulted in the loss of one patient in the analysis on the Katz scale, with an observed value of zero.

With regard to the correlation of deltas (declines) from functional status rating scales in relation to clinical variables and length of stay, Table 9 presents Spearman's correlation coefficient (r_s_), the respective descriptive level (P) and the number of cases (n) for each correlation, analyzed between deltas from the three rating scales in relation to the clinical variables and length of stay, in the full sample.

**Table 9.** Correlation of Functional Independence Measure, Katz scale, and Lawton scale deltas with clinical variables and length of stay, for the full sample (n = 31)

Variable		Delta (discharge - pre)			Delta (one month - pre)		
		FIM	Katz	Lawton	FIM	Katz	Lawton
Age (years)	*r*_s_	−0.197	−0.253	0.465	−0.380	−0.406	0.150
	P	0.29	0.17	0.008	0.035	0.023	0.42
	*n*	31	31	31	31	31	31
Extracorporeal circulation (perfusion)	*r*_s_	−0.092	−0.148	0.069	−0.061	−0.083	0.152
	P	0.64	0.44	0.72	0.75	0.67	0.43
	*n*	29	29	29	29	29	29
Extracorporeal circulation (anoxia)	*r*_s_	−0.141	−0.159	0.259	0.072	0,087	0.324
	P	0.52	0.47	0.23	0.74	0.69	0.13
	*n*	23	23	23	23	23	23
EuroSCORE	*r*_s_	−0.204	−0.182	−0.001	−0.362	−0.411	−0.234
	P	0.37	0.43	1.00	0.11	0.064	0.31
	*n*	21	21	21	21	21	21
Risk of death (%) - EuroSCORE	*r*_s_	−0.257	−0.189	−0.034	−0.339	−0.376	−0.268
	P	0.26	0.41	0.88	0.13	0.093	0.24
	*n*	21	21	21	21	21	21
APACHE II	*r*_s_	−0.339	−0.366	0.065	−0.344	−0.282	0.132
	P	0,073	0.051	0.74	0.068	0.14	0.49
	*n*	29	29	29	29	29	29
Risk of death (%) - APACHE II	*r*_s_	−0.319	−0.328	−0.027	−0.362	−0.311	0.174
	P	0.086	0.076	0.89	0.049	0.095	0.36
	*n*	30	30	30	30	30	30
Length of ICU stay (days)	*r*_s_	−0.117	−0.113	0.176	−0.235	−0.405	0.111
	P	0.53	0.55	0.34	0.20	0.024	0.55
	*n*	31	31	31	31	31	31
Length of hospital stay (days)	*r*_s_	−0.112	−0.205	0.246	−0.216	−0.371	0.163
	P	0.55	0.27	0.18	0.24	0.040	0.38
	*n*	31	31	31	31	31	31
Mechanical ventilation (min)	*r*_s_	0.253	0.216	0.295	−0.324	−0.225	−0.005
	P	0.28	0.36	0.21	0.16	0.34	0.98
	*n*	20	20	20	20	20	20

FIM = Functional Independence Measure; APACHE = Acute Physiology And Chronic Health Evaluation; ICU = intensive care unit.

## DISCUSSION

Despite the limitations of a small sample, this study highlights the vulnerability of functional status in relation to hospitalization of elderly patients undergoing cardiac surgery. Including occurrences of complications as an aggravating factor, changes in functional status can impair activities of daily living among the elderly as well as delay and compromise their postoperative rehabilitation, thus increasing the risk of disability and loss of independence.

Changes in functional status are expected within the context of hospitalization. For better identification of this impairment, we used the Functional Independence Measure, which includes items on self-care (activities of daily living) and mobility and emphasizes scores in dimensions such as communication and social cognition. Using age > 60 years as the definition for the elderly,^24^ the Katz and the Lawton scales were also used to monitor performance of activities of daily living among this population.

The dimensions of the Functional Independence Measure closely resemble those of the Barthel Index,^27^ which has shown consistent reliability and validity.^18^ They are used to quantify and monitor (in)dependence in performing activities of daily living in the hospital and home care settings, particularly in cases of stroke, but also in the rehabilitation of neurological, orthopedic and geriatric patients.^28–33^ The Barthel Index has also shown a positive correlation (P < 0.01) with the Lawton scale^7^ in relation to instrumental activities among the noninstitutionalized elderly.^34^

The patients were initially evaluated during the first month after hospital discharge, when limitations on performing some activities are expected. Although this was a short follow-up period, it was possible to see which dimensions were most affected. It is likely that a follow-up longer than one month after surgery would have better described the impact of the intervention. However, even during this short period, a slow albeit not uneventful recovery could be observed, especially given that this was an elderly population, with particular regard to the functional outcomes of autonomy and independence. Mathisen et al.^35^ demonstrated a positive change in cardiac and cognitive function, but with mixed results 12 months after coronary artery bypass grafting, particularly regarding predictive ability in relation to preoperative health status. Surgery is expected to improve not only cardiac function but also general abilities, and the procedure should not interfere with activities of daily living to the point of compromising the quality of life proposed preoperatively.

In the analysis on the three rating scales to assess the evolution of functional status, a significant change was observed over time (P = 0.0001). The three time periods differed significantly, i.e., there was a significant decline from pre-admission to hospital discharge and from pre-admission to one month after discharge, and a significant increase from hospital discharge to one month after discharge, as shown in Tables 3, 4 and 5.

In order to study the relevance of hospitalization-related complications to changes in functional status, from a gerontological perspective, the sample was analyzed in subgroups with and without complications. In considering quality of life in old age and the context of rehabilitation, factors going beyond autonomy and independence are involved, such as recovery of social roles, formal and informal support, environmental safety and health status.^36^ During hospitalization, complications were observed in 18 patients (58%) (Table 2). For the elderly, these intercurrent events may have a great impact and significantly affect not only their functional status but also their home environment, because of the possible need for further healthcare and changes such as bringing in caregivers or institutionalization.^37^

Delirium, for example, is a prevalent complication among elderly patients during the postoperative period following cardiac procedures (23–27%). This contributes not only towards extending the length of hospital stay, but also as an important predictor of long-term cognitive impairment, thus increasing the risk of death. Knowledge of and control over risk factors such as hypertension, smoking, atrial fibrillation and those relating to coronary artery bypass surgery (like infection and blood transfusion during surgery) may contribute towards reducing the morbidity and mortality that result from delirium.^38,39^ In the case of urinary tract infection, which is the most prevalent infectious complication, preventive measures should be adopted in order to avoid inflammatory responses such as sepsis, which increase the severity of the disease, compromise patients' outcomes and postpone discharge.^40^ In addition, care with and controlled use of blood derivatives should be based on safety recommendations.^41^

Repeated-measurement analysis of variance showed significant changes in the three rating scales for both subgroups, with and without complications (P = 0.0001). A significant decrease from pre-admission to hospital discharge and a significant increase from hospital discharge to one month after discharge were identified in both subgroups, but only the subgroup with complications showed a significant decline from pre-admission to one month after discharge, as shown in Tables 6 and 7 and Figure 1. On the Lawton scale, a significant decrease was also observed from pre-admission to one month after discharge, as shown in Table 8. However, no significant changes were observed in the Functional Independence Measure (P = 0.085), Katz scale (P = 0.12) and Lawton scale (P = 0.74) over time between the subgroups at the 5% level, i.e. the two subgroups evolved similarly. Nevertheless, we can affirm that, according to the Functional Independence Measure and the Katz scale, the subgroup without complications showed a trend toward recovery (of pre-admission functional status), one month after hospital discharge. This trend underscores the perspective that mental and physical rehabilitation may be promoted after hospital discharge, especially among elderly patients with complications.^5,11,18,19,33,39^

In the analysis on clinical variables and length of stay (Table 9), there was a significant direct correlation between Lawton scale delta (hospital discharge/pre-admission) and age (r_s_ = 0.465, P = 0.008, n = 31). This means that the older the patient was, the higher the expected value of Lawton scale delta (decline) for hospital discharge in relation to pre-admission would be, thus demonstrating the vulnerability of older patients to perform instrumental activities of daily living after hospital discharge.^6,7,18,34^

Regarding length of extracorporeal circulation and duration of mechanical ventilation, there was no significant correlation in either delta, probably because of their length. However, regarding APACHE II and EuroSCORE severity scores, there was a trend toward a significant indirect correlation in both deltas. For the Katz scale delta only (one month after discharge/pre-admission), EuroSCORE showed the following values: r_s_ = −0.411, P = 0.064 (n = 21). This means that the higher the risk score value was, the lower the Katz scale delta (decline) between one month after discharge and pre-admission would be. Patient, cardiac, and surgical factors correspond to the operative risk evaluation. In this case, patients' high risk scores corresponded to a Katz scale value (one month after discharge) closer to baseline values (pre-admission), with successful recovery of their ability to perform daily living activities.^5,22,35^

Concerning APACHE II, there was also a significant indirect correlation with deltas (hospital discharge/pre-admission) from the Functional Independence Measure (r_s_ = −0.339, P = 0.073, n = 29) and from the Katz scale (r_s_ = −0.366, P = 0.051, n = 29). This means that the higher the severity score value was, the lower the Functional Independence Measure and Katz scale deltas (declines) between hospital discharge and pre-admission would be. That is, patients with greater severity of disease were likely to return to their pre-admission functional status, probably due to successful recovery. However, they were also likely to need more than one month after hospital discharge to return to baseline values (pre-admission) in this case, as measured by the Functional Independence Measure delta (one month after discharge/pre-admission): r_s_ = −0.344, P = 0.068, n = 29; for APACHE II.^4,5,14,19,21^

Regarding ICU and overall hospital lengths of stay, there was a significant indirect correlation with Katz scale delta (one month after discharge/pre-admission) (r_s_ = −0.405; P = 0.024, n = 31; and r_s_ = −0.371, P = 0.040, n = 31, respectively, for ICU stay and hospital stay). Patients who stayed longer in both the ICU and the hospital would possibly need more than one month after hospital discharge to return to baseline values corresponding to their pre-admission functional status, as measured by the Katz scale. This fact demonstrates that a long hospital stay may pose a risk for the recovery of autonomy and independence to perform daily living activities among the elderly.^5,18,38,39^

It may be asked, within this context, whether the most critical patients are kept longer in both the ICU and the hospital, in order to achieve full recovery, such that they are only discharged after significant improvement in clinical status. This would contribute towards the delta (decline) values observed between hospital discharge/pre-admission and between one month after discharge/pre-admission from the functional status rating scales, taking into account the risks of a long stay.

Despite the small sample size and study period, the Functional Independence Measure proved to be a promising tool, since it took into consideration factors going beyond activities of daily living, such as social behavior and cognition, in following up this cohort of patients. Such findings are likely to be replicated with a larger sample, and a longer follow-up period should also be taken into consideration.

## CONCLUSION

The functional status of elderly coronary patients undergoing coronary artery bypass showed significant changes, as assessed by the three functional rating scales between the time points of pre-admission, hospital discharge and one month after discharge. Similarly, this change was observed when comparing the subgroups with and without complications.

Age, risk and severity scores and length of stay were significantly correlated with the deltas (declines) relating to the evolution of the functional status rating scales used in this study. However, this was not observed for length of extracorporeal circulation or mechanical ventilation.

## ACKNOWLEDGEMENTS

The authors are grateful to Rosângela Aparecida Martins for performing the statistical analysis; and to Humberto Villacorta MD, MSc, PhD, and André Miguel Japiassú MD, MSc, PhD, for providing technical assistance

Partial results from this study were presented in the following scientific events: South American Congress on Geriatrics and Gerontology (Porto Alegre, Brazil, 2009); Brazilian Congress of Critical Care Medicine (São Paulo, Brazil, 2009, and Brasília, Brazil, 2010); Annual Scientific Meeting of the American Geriatrics Society (Orlando, Florida, United States, 2010); and Brazilian Congress of Geriatrics and Gerontology (Belo Horizonte, Brazil, 2010)
